# Use of Physiologically Based Pharmacokinetic (PBPK) Modeling for Predicting Drug-Food Interactions: an Industry Perspective

**DOI:** 10.1208/s12248-020-00508-2

**Published:** 2020-09-27

**Authors:** Arian Emami Riedmaier, Kevin DeMent, James Huckle, Phil Bransford, Cordula Stillhart, Richard Lloyd, Ravindra Alluri, Sumit Basu, Yuan Chen, Varsha Dhamankar, Stephanie Dodd, Priyanka Kulkarni, Andrés Olivares-Morales, Chi-Chi Peng, Xavier Pepin, Xiaojun Ren, Thuy Tran, Christophe Tistaert, Tycho Heimbach, Filippos Kesisoglou, Christian Wagner, Neil Parrott

**Affiliations:** 1 DMPK and Translational Modeling, AbbVie Inc., North Chicago, Illinois USA; 2 Global DMPK, Takeda Pharmaceutical Co., Ltd., San Diego, California USA; 3 Drug Product Technology, Amgen, Thousand Oaks, California USA; 4 Modeling & Informatics, Vertex Pharmaceuticals, Boston, Massachusetts USA; 5 Pharmaceutical R&D, Formulation & Process Sciences, F. Hoffmann-La Roche Ltd., Basel, Switzerland; 6 Computational & Modelling Sciences, Platform Technology Sciences, GlaxoSmithKline R&D, Ware, Hertfordshire, UK; 7 Clinical Pharmacology and Safety Sciences, R&D, AstraZeneca, Cambridge, UK; 8 Pharmacokinetic, Pharmacodynamic and Drug Metabolism-Quantitative Pharmacology and Pharmacometrics (PPDM-QP2), Merck & Co, Inc., West Point, Pennsylvania USA; 9 Department of Drug Metabolism and Pharmacokinetics, Genentech, South San Francisco, California USA; 10 Formulation Development, Vertex Pharmaceuticals, Boston, Massachusetts USA; 11 Present Address: Formulation Development, Cyclerion Therapeutics Inc., Cambridge, Massachusetts USA; 12 Chemical & Pharmaceutical Profiling, Novartis Institutes for Biomedical Research, Cambridge, Massachusetts USA; 13 Department of Pharmacokinetics and Drug Metabolism, Amgen Inc., Cambridge, Massachusetts USA; 14 Pharmaceutical Sciences, Roche Pharmaceutical Research and Early Development, Roche Innovation Center, Basel, Switzerland; 15 Present Address: Drug Metabolism and Pharmacokinetics, Theravance Biopharma, South San Francisco, California USA; 16 New Modalities and Parenteral Development, Pharmaceutical Technology & Development, Operations, AstraZeneca, Macclesfield, UK; 17 Modeling & Simulation, PK Sciences, Novartis Institutes of Biomedical Research, East Hanover, New Jersey USA; 18 Computational & Modelling Sciences, Platform Technology Sciences, GlaxoSmithKline R&D, Collegeville, Pennsylvania USA; 19 Pharmaceutical Sciences, Janssen Research & Development, Beerse, Belgium; 20 PBPK & Biopharmaceutics, Novartis Institutes of Biomedical Research, Wayne, New Jersey USA; 21 Pharmaceutical Sciences, Merck & Co., Inc., Kenilworth, New Jersey USA; 22 Pharmaceutical Technologies, Chemical and Pharmaceutical Development, Merck Healthcare KGaA, Darmstadt, Germany; 23 Pharmaceutical Sciences, Roche Pharmaceutical Research and Early Development, Roche Innovation Center, Basel, Switzerland

**Keywords:** drug-food interaction, food effect, modeling and simulation, PBBM, PBPK

## Abstract

The effect of food on pharmacokinetic properties of drugs is a commonly observed occurrence affecting about 40% of orally administered drugs. Within the pharmaceutical industry, significant resources are invested to predict and characterize a clinically relevant food effect. Here, the predictive performance of physiologically based pharmacokinetic (PBPK) food effect models was assessed via *de novo* mechanistic absorption models for 30 compounds using controlled, pre-defined *in vitro*, and modeling methodology. Compounds for which absorption was known to be limited by intestinal transporters were excluded in this analysis. A decision tree for model verification and optimization was followed, leading to high, moderate, or low food effect prediction confidence. High (within 0.8- to 1.25-fold) to moderate confidence (within 0.5- to 2-fold) was achieved for most of the compounds (15 and 8, respectively). While for 7 compounds, prediction confidence was found to be low (> 2-fold). There was no clear difference in prediction success for positive or negative food effects and no clear relationship to the BCS category of tested drug molecules. However, an association could be demonstrated when the food effect was mainly related to changes in the gastrointestinal luminal fluids or physiology, including fluid volume, motility, pH, micellar entrapment, and bile salts. Considering these findings, it is recommended that appropriately verified mechanistic PBPK modeling can be leveraged with high to moderate confidence as a key approach to predicting potential food effect, especially related to mechanisms highlighted here.

**Electronic supplementary material:**

The online version of this article (10.1208/s12248-020-00508-2) contains supplementary material, which is available to authorized users.

## INTRODUCTION

For orally administered drugs, the consumption of food at the time of drug administration can alter absorption (1). Food effect is widespread, and over 40% of the orally administered drugs approved by the FDA or EMA in the last 10 years were reported to have altered pharmacokinetics (PK) by food (2). Therefore, health authorities expect sponsors to characterize food or meal effects prior to approval (3,4). While a food effect assessment is typically performed with a standardized high-fat meal, in certain cases, evaluation of different meal types (i.e., varying macro and calorie contents) may be recommended. If food does not have a clinically significant impact on PK, then sponsors can conduct pivotal trials without regard to food and labeling can state that the drug may be taken with or without food. However, in the case of a clinically meaningful PK effect, a specific recommendation will be made for drug administration; for example, a drug can only be given under fasted conditions or taken with a meal to maximize drug absorption.

Recently updated FDA guidance on the conduct of food effect studies describe, in detail, the clinical study design, data analysis, and labeling recommendation. Notably, neither the recent FDA guidance nor guidance from other agencies has mentioned the utility of mechanistic studies of food effect using *in vitro* and *in silico* models. However, within the pharmaceutical industry, significant resources are often invested to anticipate, characterize, and mitigate a food effect, since clinical studies alone do not provide the mechanistic insights needed to predict and understand food effects (2). Recent reviews have covered the multiple mechanisms for food effect (5) and the tools available to understand them (6). Among them, PBPK modeling has gained critical attention for the prediction of food effects using the advanced absorption models in commercial software platforms such as GastroPlus™ and Simcyp® (7–9). However, despite these examples, the health authorities still lack confidence in these predictions (8,10). This may, in part, be due to a lack of best practices for consistent modeling strategy, as well as, lack of a prospective approach to evaluating the success of food effect predictions.

Therefore, this work aims to assess the predictive performance of PBPK food effect models and to provide recommendation for best practice. Instead of relying on previously published models, the Food Effect PBPK IQ Working Group generated de novo mechanistic absorption models for 30 compounds using physicochemical and *in vitro* data generated in accordance with pre-defined methodology. Furthermore, a decision tree is proposed for model verification and optimization, which was strictly followed within the working group. Thus, this work provides a well-controlled assessment of PBPK food effect modeling by minimizing confounding factors, such as inconsistent data generation, subjective model verification/optimization, and variable modeler experiences.

## Materials and Methods

To reduce methodological bias in PBPK input parameters, this work mostly used permeability, solubility, surface pH, and dissolution data generated using pre-defined methods by this working group. However, if literature data used comparable methods, values were not re-measured. This information is indicated in Table I.

**Table I:** Summary of the Food Effect Direction, Dose at Observed Food Effect, and Physicochemical and Absorption Properties of the 30 Modeled Compounds

Compound	Maximum dose in food effect study	Food effect	Molecular weight^*1*^ (g/mol)	cLogD^*2*^	pKa	Apparent permeability (× 10^−6^ cm/s)	Effective permeability (× 10^−4^ cm/s)^*3*^	Solubility at pH 6.5 (μg/ml)	FaSSIF solubility (μg/ml)	FeSSIF solubility (μg/ml)	BCS	Reference
Alectinib	600	Positive	482.6	4.8	7.05b	n/a	2.5^^^		23	77	II	(11)
Amiodarone	600	Positive	645.3	6.5	5.90b	n/a	0.9^^^	2	472	784	II	(12)
Aprepitant	100	Positive	534.4	5.2	2.45b, 9.15a	13 (MDCK)	2.4	0.7	5.4	92.4	II/IV	(13,14)
Cimetidine	200	None	252.3	− 0.2	6.90b	5.4 (Caco-2)^^^	1.278		22,000	21,500	III	Simcyp value for P_eff_
Clarithromycin	500	None	748.0	2.2	8.99b	5 (MDCK)	0.3		132	1656	II	
Dabrafenib	150	Negative	519.6	4.6	2.2b, 6.6a	84 (MDCK)	5.5	1	6.2^^^	6.8^^^	II	(15)
Danazol	100	Positive	337.5	3.5	Neutral	4.2 (MDCK)	1.0^^^	0.69	7270	52,600	II	(16–18)
Danirixin	50	Negative	441.9	1.8	4.80a, 8.10b	n/a	0.9^^^		459	724	II	GSK in-house data
d-Sotalol	160	None	272.4	− 2.1	8.28a, 9.72b	n/a	1.2^^^	15,800^^^	n/a	n/a	III	(19)
Etoricoxib	120	Negative	358.8	2.8	4.5b	52.3 (Caco-2)^^^	4.8	67^^^	67^^^	95^^^	II	(20)
Fluoxetine HCl	40	None	309.3	1.8	9.8b	1.69 (MDCK)	1.0^^^	1533	6400	1720	I	
Furosemide	40	Negative	330.7	− 1.3	3.9a	n/a	0.5^^^	5188^^^	3201^^^	684^^^	IV	(21–23)
Imatinib	400	None	493.6	3.5	3.73b, 8.07b	4.7 (MDCK)	1.1	113	220^^^	2210^^^	II	(24)
Isoniazid	300	Negative	137.1	− 0.7	1.80b, 3.50b	16 (Caco-2)^^^	4.0	128,000	n/a	n/a	I	(25)
Itraconazole	100	Positive	705.6	7.3	4.28b	n/a	9.9		1120	3620	II	
Ivacaftor	150	Positive	392.5	4.3	9.40a, 11.60a	11.9 (Caco-2)^^^	1.2	0.5^^^	53^^^	550^^^	II/IV	Vertex in-house data
Metoprolol	100	Positive	267.4	− 0.5	9.75b	n/a	1.3^^^	17,100	n/a	n/a	I	(26)
Nefazodone HCl	200	Negative	470.0	4.5	6.50b	11 (Caco-2) ^^^	2.2	5378	1393	3100	II	
Nelfinavir mesylate	1250	Positive	567.8	4.1	6.0b	1.3 (MDCK)	0.7	41.4^^^	22^^^	243^^^	II/IV	(27,28)
Nifedipine	10	None	346.3	1.8	Neutral	23.5 (Caco-2)^^^	12.5	9.2^^^	17.1^^^	56.2^^^	II	(29,30)
Oseltamivir	150	None	312.4	− 0.7	7.70b	n/a	1.5^^^	25,000^^^	n/a	n/a	III	(31)
Panobinostat	20	None	349.4	0.7	8.35a, 9.0b	11 (Caco-2)^^^	2.3	261^^^	140^^^	230^^^	II	Novartis in-house data
Pazopanib	800	Positive	437.5	3.5	2.1b, 6.4b	16.9 (Caco-2)^^^	2.3	0.5	1.2	2.8	II/IV	(32)
Phenytoin	300	Positive	252.3	1.2	8.06a	51 (MDCK)	4.0	31^^^	39.6^^^	49.5^^^	II	(33)
Telaprevir	750	Positive	679.8	2.6	Neutral	4.4 (Caco-2)^^^	1.4	85	120	80	II	Vertex in-house data
Tezacaftor	50	None	520.5	3.4	Neutral	4.2 (Caco-2)^^^	2.5	110^^^	119^^^	4783^^^	II	Vertex in-house data
Trospium IR	30	Negative	392.5	− 0.5	+Charged	n/a	0.1 (0.005 to 0.5)	780	n/a	n/a	III	(34,35)
Trospium XR	60	Negative	392.5	− 0.5	+Charged		0.1 (0.005 to 0.5)	780	n/a	n/a	III	
Venetoclax	100	Positive	868.4	6.5	3.40a; 10.3b	n/a	1.0^^^	6	0.0337	26.4	IV	(7)
Zidovudine	300	Negative	267.2	− 0.4	9.70b	n/a	3.7^^^		> 10	> 10,000	III	
Ziprasidone HCl	80	Positive	412.9	4.1	6.58b	12.3 (Caco-2)^^^	2.3	1	4	13	II	(36)

For highly soluble and/or hydrophilic compounds, the impact of bile salts on the solubility was considered negligible and FaSSIF/FeSSIF data was not reported

*n/a*, not available

^*1*^Molecular weight of active ingredient only. The counterion of salts is excluded from the molecular weight

^*2*^Log10 of the octanol/water distribution coefficient at pH 7.4 as calculated with the OpenEye software

^*2*^Effective jejunum permeability used in the PBPK model

^^^Data was not generated within this working group. Source of data provided in the reference column

### Compound Selection

**Fig. 1:**
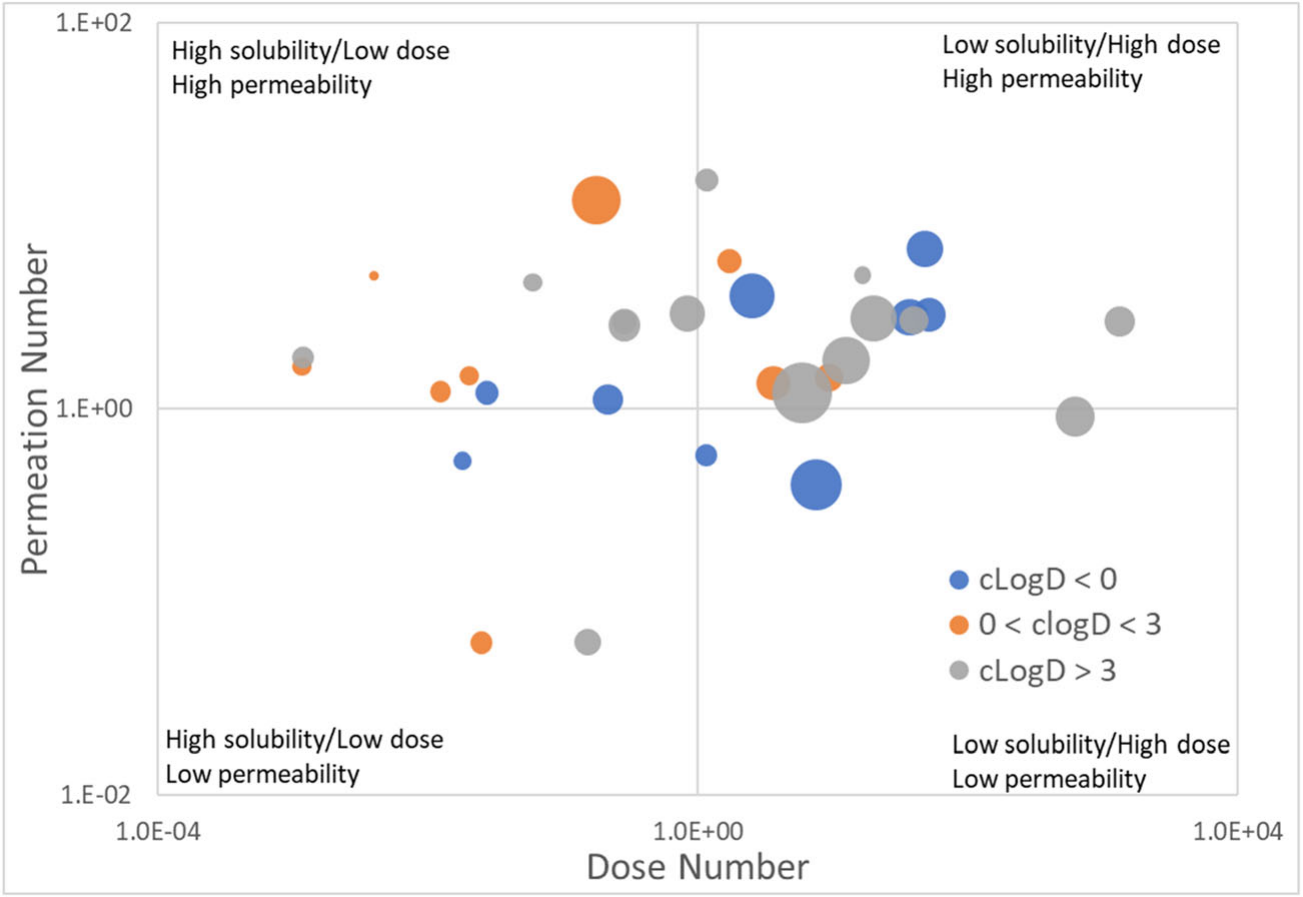
Physicochemical properties of the 30 modeled compounds. The compounds selected cover a range of solubility, permeability, molecular weight, and lipophilicity. A compound’s unitless dose number is calculated as the maximum dose administered in the food effect study in mg, divided by the FaSSIF or buffer solubility in mg/ml, and divided by an approximate small intestine fluid volume of 500 ml. A dose number greater than 1 indicates low solubility or a high dose while a dose number less than one indicates high solubility or low dose. The unitless permeation number is calculated as the effect jejunum permeability multiplied by the surface-to-volume ratio of the small intestine assuming a 1.75 cm cylindrical radius, multiplied by the small intestine transit time assumed to be 3 h. A permeation number greater than one indicates high permeability while a permeability less than 1 indicates poor permeability. The size of the markers is proportional to the active ingredient’s molecular weight. The color encodes the calculated lipophilicity

A comprehensive compound list with clinically observed PK changes in the presence of food was collated through a detailed literature search and curation (Supplementary Table 1). The information collected included the outcome of a food effect study, the compound type (i.e., acid, base, or ampholyte), and proposed mechanism of food effect. To focus on absorption-related mechanisms of food effect and to reduce variability in modeling where there is low confidence in the disposition of a given compound, all compounds lacking clinical intravenous (IV) PK data or population PK-based data, as well as compounds with high hepatic extraction, were excluded from this list. Furthermore, prodrugs and compounds whose absorption is known to be limited by active transport were excluded, though these compounds are not expected to make up a large subset of clinical compounds displaying food effect (Supplementary Table 1). The compound list was subsequently refined to 30 compounds for final modeling and analysis while ensuring equal distribution of compound, BCS, and food effect type (Table I, Fig. 1). Food effect (FE) type was defined based on AUC and/or *C*_max_ ratios of fed to fasted using BE criteria (i.e., within 0.8–1.25). FE definitions were based on the drug label and set to positive if the ratio of fed > fasted, negative if ratio fasted > fed, or none if no significant change in AUC and *C*_max_ with food.

### Permeability Measurement in MDCK Cells

Wild-type Madin-Darby canine kidney (MDCK-WT) cell line was obtained from NKI (Amsterdam, The Netherlands) and modified to knockdown endogenous canine P-glycoprotein (P-gp). Permeability through a cell monolayer was determined with a Transwell™ system. Cells were plated on the apical side of 96-well Transwell plates 4–7 days prior to the experiment and were cultured at 37°C under a 5% CO_2_ atmosphere. All compounds were dissolved in Hanks’ balanced salt solution (HBSS) plus 80 mM Lucifer Yellow (LuY) and 10 μM cyclosporin A, which was added to the apical wells. The corresponding receiver (basolateral) wells were filled with HBSS plus 10 μM cyclosporin A, a P-gp Inhibitor.

Permeation rates of the compounds, including reference compounds, were measured in the apical-to-basolateral (AB) direction. The donor and receiver wells were sampled immediately after application of the compound to the donor well to determine baseline concentrations, and again after 1 h. Quantification was done using high-performance liquid chromatography combined with mass spectrometry (HPLC-MS/MS) analysis and monolayer integrity was verified by analyzing the receiver samples for LuY fluorescence in a plate reader. Control compounds were run in parallel to test compounds and were used to scale the apparent permeability (*P*_app_ × 10^−6^ cm/s) to an effective human permeability (*P*_eff,man_ × 10^−4^ cm/s) using the software’s built-in calibration curve (21).

### Solubility Measurement in Aqueous Buffer Solutions and Biorelevant Media

The solubility of the drug substances was determined in biorelevant media as well as in aqueous buffers at different pH. Fasted state simulated gastric fluid (FaSSGF) (37), fasted state simulated intestinal fluid (FaSSIF-V2) (38), and fed state simulated intestinal fluid (FeSSIF-V2) (39) were prepared according to the instructions provided by Biorelevant (Biorelevant.com Ltd., London, UK). Hydrochloric acid pH 2, citrate buffer pH 4, and phosphate buffer pH 7, as well as additional buffer solutions if required, were prepared according to the standard buffer solutions described in the United States Pharmacopeia (USP) [USP 41, buffer solutions, 5748–5749]. For neutral compounds, solubility was determined at pH 2, pH 4, and pH 7. For ionizable compounds, two additional solubility data points, one pH unit above and below the pKa value(s), were collected.

Excess of drug substance was equilibrated in the media on a magnetic stirrer (200 rpm) at 37°C (biorelevant media) and at room temperature (aqueous buffer). The concentration of dissolved drug and the medium pH were determined after 1, 2, 6, and 24 h. The equilibrium solubility was interpreted as the concentration measured after a plateau was reached and was at the latest measured after 24 h. For freely soluble compounds, the extent of solubilization was measured only up to 10 mg/mL.

### PBPK Modeling Approach

**Fig. 2:**
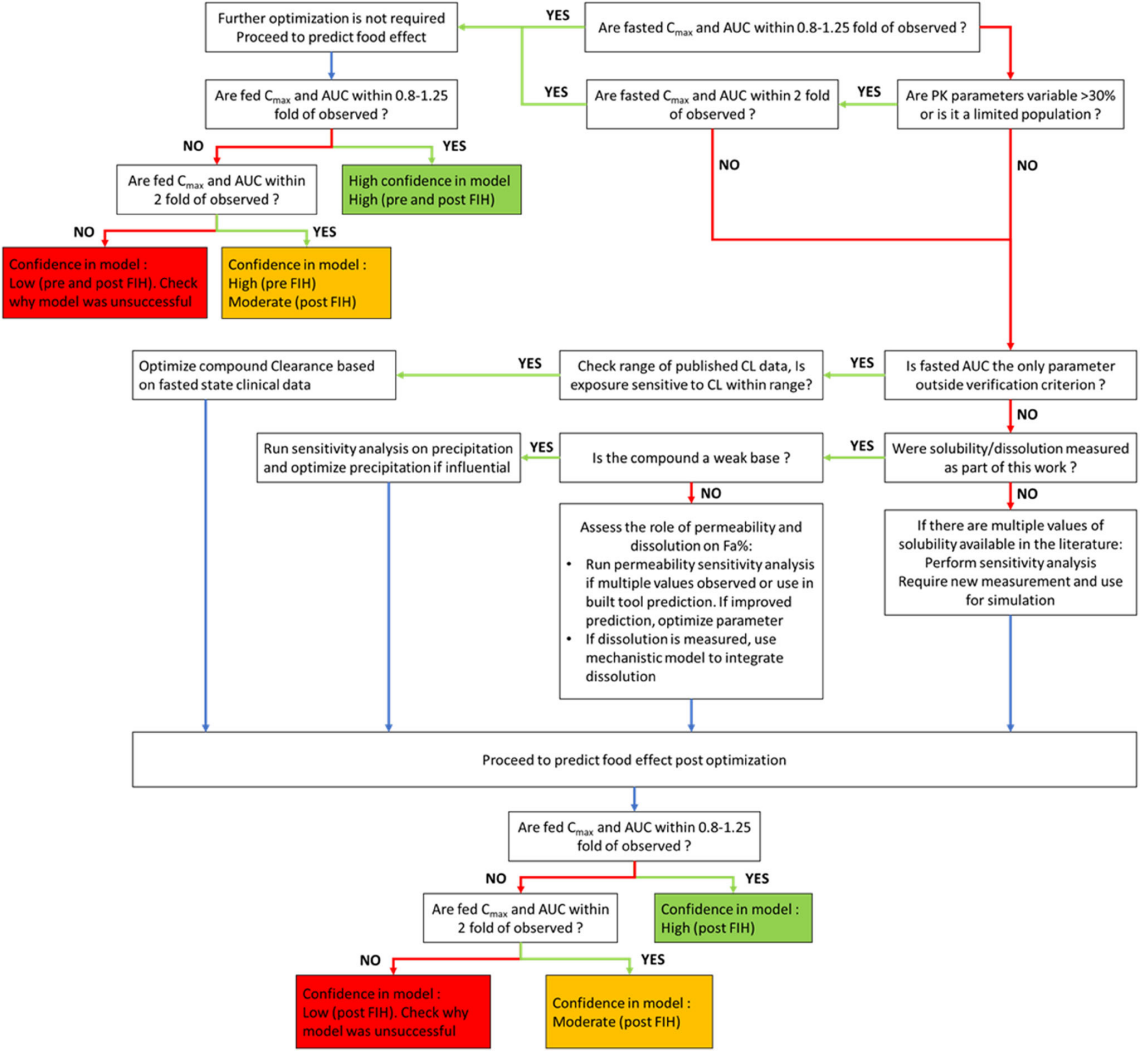
Decision tree for the verification and optimization of food effect projections using PBPK. This decision tree was utilized by all modelers working on this initiative to verify and, if necessary, optimize their models using an aligned and consistent approach. Confidence categories were defined based on the outcome of this workflow after an independent review of the model outcome and verification. A summary of the outcome of PBPK modeling based on this decision tree for the 30 compounds is provided in Table II

An aligned decision tree was defined by working group members prior to modeling, as outlined in Fig. 2. In short, PBPK models were built for all compounds in Simcyp V17.1 (Certara, USA, Inc.) and/or GastroPlus V9.5 (Simulations Plus, Inc.). A software comparison was not the aim of this working group. However, if the results of the two software platforms (i.e., model 1 vs. model 2) showed any large discrepancies, this was reported (Table II, footnotes), and where possible, the underlying mechanisms were investigated and described (Table III, Fig. 5). For GastroPlus, individual, population-representative simulations were conducted as best practice. For Simcyp, all simulations were run in the healthy volunteer population using the default system parameters and with the clinical trial design and doses matched to the reported studies. Published and measured values for physicochemical properties, permeability, solubility, and dissolution were utilized as input parameters, respectively, to build mechanistic, bottom-up models for absorption. In order to reduce the uncertainty and variability and narrow the analysis of food effect predictions to absorption-related mechanisms, clearance and disposition were modeled based on published clinical IV PK and/or population PK data. This was not done because IV data is required or recommended by the working group for PBPK model success, but rather to simplify the modeling approach and subsequent analyses, such that model outcomes could be interpreted in the context of absorption parameters only. Furthermore, the focus of this work was to study FE related to absorption mechanisms and not, for example, hepatic first pass or metabolism changes.

**Table II:**
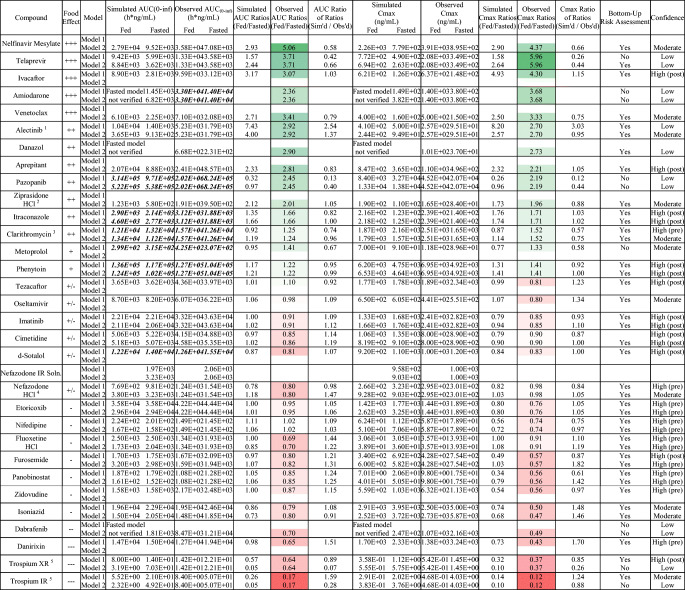
Summary of the Outcome of Food Effect PBPK Modeling for 30 Compounds and the Associated Confidence in the PBPK Food Effect (FE) Prediction and Risk Assessment. The Color Coding Represents the Food Effect Direction with Green and Red Signifying Positive and Negative Food Effect, Respectively

Bold italicized text indicates AUC(0-t), not AUC(0-inf)

^*1*^The model-specific discrepancy in confidence for alectinib is not currently well understood

^*2*^Although ziprasidone qualifies as high confidence given AUC and *C*_max_ ratios of ratios which fall within bioequivalence criteria, the simulated, fed-state plasma concentration-time profile poorly captured observed data. As such, ziprasidone was qualified as moderate confidence

^*3*^Although clarithromycin model 2 demonstrated superior food effect prediction accuracy, model 2 required optimization to capture fasted clinical data. As model 1 utilized a purely bottom-up approach, confidence in that model is higher

^*4*^Simulation of clinical nefazodone concentration-time data initially resulted in overprediction, possibly explained by partial gastric emptying in vivo. Model 1 but not model 2 incorporated partial gastric emptying, explaining the final model-specific discrepancy in confidence

^*5*^The use of different methods to optimize individual segmental Peffs between models 1 and 2 may explain the model-specific discrepancy in confidence for trospium IR and XR formulations

**Table III:**
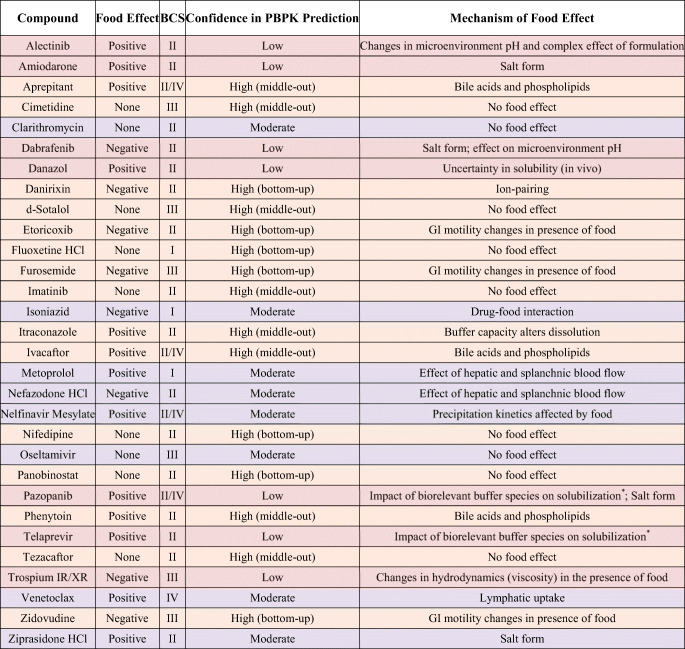
Summary of the Proposed Mechanism of Food Effect and the Associated Confidence Category in the PBPK Prediction of Food Effect. Color Coding Indicates Confidence in the PBPK Food Effect Prediction; Green: High; Yellow: Moderate; Red: Low

*Specialized biorelevant media required to capture food effect

The decision tree outlined specific criteria for a model to be considered verified, as well as potential steps to optimize a model where necessary (Fig. 2). The decision tree was followed by all modelers and used to determine the degree of success in predicting food effect. Parameters that were optimized were limited to clearance, precipitation time, and/or permeability. Due to uncertainty in the bio-relevance of *in vitro* solubility data, solubility was not optimized once the relevant solubility input was evaluated by comparing the different *in vitro* measured solubility values. In some cases where the decision tree did not lead to a successful model even after optimization, additional steps were taken to optimize the model to enable hypothesis testing; these examples are further discussed in an accompanying manuscript in this issue (e.g., discussion on pazopanib).

### Confidence Criteria

PBPK models were developed based on the decision tree outlined in Fig. 2, initially using a bottom-up approach and subsequently using a middle-out approach for cases where verification based on the decision tree criteria was not successful. Compounds were assigned to a pair of modelers with one modeler building the model and the other reviewing it for accuracy and goodness of fit. Success was defined based on visual inspection of the PK profile overlay (i.e., if there was a *T*_max_ or *C*_max_ shift), as well as quantitative assessment of *C*_max_ and AUC ratios (verification range defined in Fig. 1 and described in more detail below).

Model performance was evaluated in the context of the stage of drug development (i.e., purely bottom-up vs. middle-out) using two key criteria: confidence in predicting the likelihood of food effect (i.e., risk assessment) and confidence in predicting the direction and extent of food effect.

The first criterion was assessed using a qualitative yes/no categorization in answer to the question: was the food effect captured correctly in the absence of model optimization with clinical data?

The second criterion was quantitative in nature and involved evaluation of observed versus predicted AUC and *C*_max_ ratios of fasted and fed.

When the bottom-up model could accurately capture the fasted and fed PK parameters and profile within 2-fold of observed, and visual inspection indicated good overlay of the PK profiles without the need for optimization of absorption parameters, the model was considered to have high confidence with respect to bottom-up success (confidence category: high confidence bottom-up). Second, where the bottom-up model could accurately capture the fasted and fed PK parameters and profile within 0.8–1.25 range, but only after optimization of absorption parameter(s) as defined in the decision tree, the model was considered high confidence with respect to middle-out success, e.g., for informing food effect of new formulations and dose strengths (confidence category: high confidence middle-out). Third, where the model could capture the fasted and fed PK parameters and profile following optimization using fasted data, though it fell outside the conservative criteria defined above, but within 2-fold of observed PK parameters, the model was considered to have moderate confidence. Finally, where the model failed to capture the fasted and/or fed PK parameters and profile even after optimization as described in the decision tree, it was categorized as low confidence (Fig. 2). While modeling the latter subset of compounds using a broad, pre-defined decision tree around optimization was not found to be suitable, deviating from the general workflow helped improve the accuracy of some of the models; these examples are captured in an accompanying manuscript in this issue focusing on low confidence predictions.

## RESULTS

**Fig. 3:**
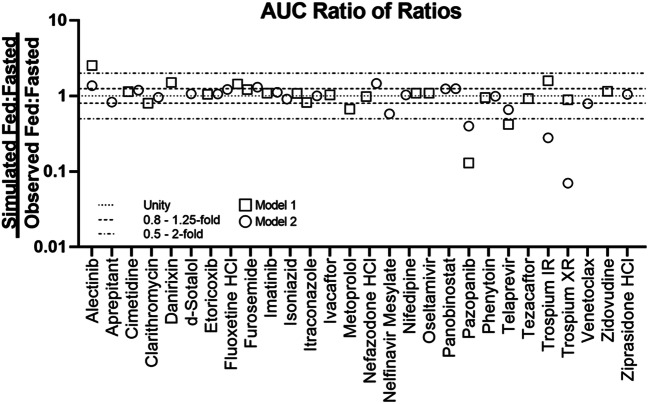
AUC ratio of ratios for the modeled compounds. Models 1 and 2 refer to the two software programs used for prediction. Where confidence did not agree between the two software, the outcome from the model with lower confidence was used to assign confidence in the prediction

**Fig. 4:**
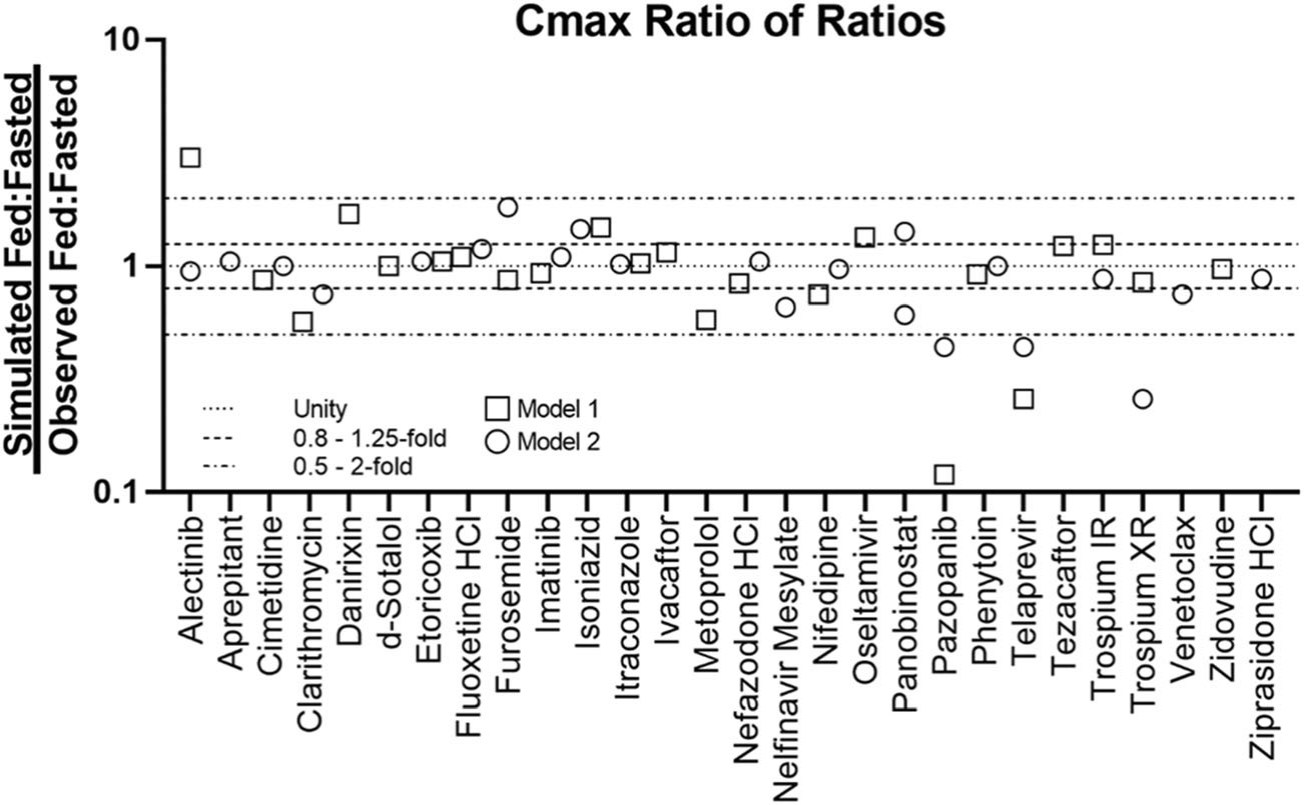
*C*_max_ ratio of ratios for the modeled compounds. Models 1 and 2 refer to the two software programs used for prediction. Where confidence did not agree between the two software, the outcome from the model with lower confidence was used to assign confidence in the prediction

**Fig. 5:**
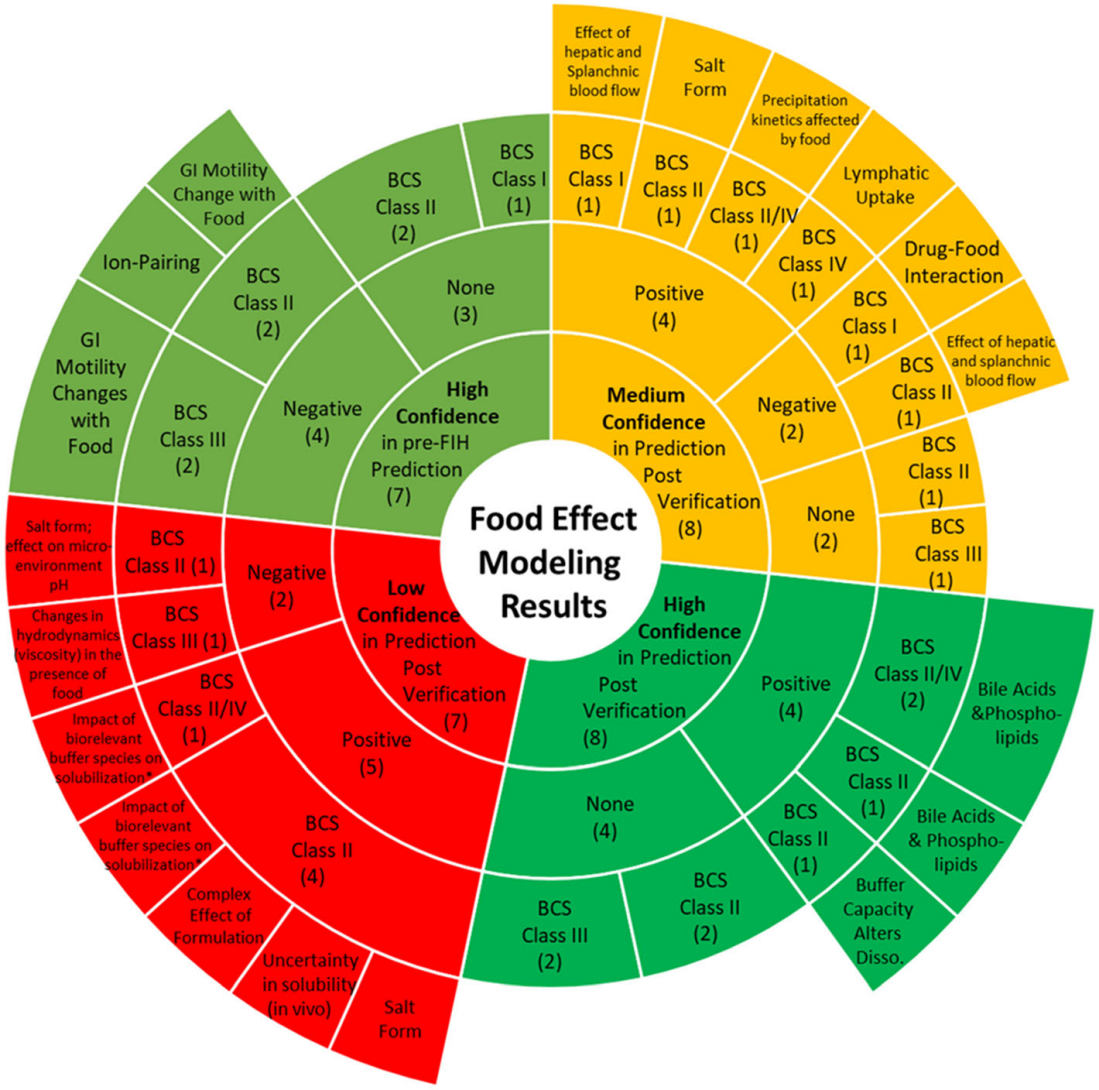
Proposed mechanisms of food effect and their association with confidence in PBPK modeling for 30 modeled compounds. The inner layer of the plot depicts the confidence category, followed by direction of food effect in the second row, the BCS class in the third row, and the mechanism of food effect in the fourth row. The numbers in the first to third row indicate the number of compounds (out of 30) that fall in each category. More details around compound name and mechanism are provided in Table III

Based on the exclusion criteria described above, 30 compounds were selected for modeling. The selected compounds showed diverse properties and included 13 compounds with positive food effect, 8 compounds with negative food effect, and 9 compounds with no food effect. Of the 30 compounds modeled, 24 of the models could correctly capture the likelihood of food effect without any optimization, this is indicated by a yes/no on Table II (i.e., risk assessment) (Table II). Furthermore, the impact of food on PK was predicted with high, moderate, and low confidence for 15, 8, and 7 compounds, respectively (Table II, Figs. 3 and 4). There was no clear correlation between the prediction confidence and BCS category and/or food effect type. However, an association could be demonstrated with the key mechanism(s) driving the food effect (Fig. 5, Table III). High confidence in PBPK prediction of food effects was typically observed for compounds where the mechanism of food effect was related to physiology, including changes in the gastrointestinal (GI) luminal fluids, fluid volume, motility, pH, ion pairing, and bile salts. Low confidence in prediction was associated with food effects related to drug formulation interactions with the intestinal microenvironment, specifically with respect to salts and weak bases such that the model and/or the biorelevant media used could not capture the dynamic effect of the drug on its microenvironment. Low confidence in modeling was also observed with food effects related to fed-state hydrodynamics (e.g., GI fluid viscosity) and food-drug/micelle-drug interactions where standard *in vitro* assays are not able to characterize the food effect mechanistically.

Three examples from the different confidence categories have been highlighted here. For category I, a high confidence example for bottom-up application demonstrates successful verification using a purely bottom-up approach for modeling absorption and related FE. Category II exemplifies the successful verification following a middle-out approach using the optimization decision tree, identified as high confidence for post-FIH applications. Finally, an example from category III shows a failed verification following optimization using the outlined strategy. Here, confidence in using a general workflow for the prediction of food effect is low and increased confidence will depend on a case-by-case evaluation of more predictive *in vitro* and modeling methodologies.

### PBPK Predictions to Waive Food Effect Studies—Bottom-Up Application

#### Case Example: Nifedipine (High Confidence)

Nifedipine is a poorly soluble, non-ionizable compound with no food effect reported on the extent of absorption when administered as a 10 mg IR soft gelatin capsule, although the rate of absorption decreased with food (40).

The capsule contains nifedipine dissolved in an organic solvent (polyethylene glycol 400 and peppermint oil) and thus behaves like a solution.

The mechanistic, bottom-up absorption model was built using previously published solubility data (29). Precipitation time was kept at the default. The apparent Caco-2 permeability was taken from the literature (Gertz, Harrison *et al.* 2010) and converted into a human effective permeability, using the software’s built-in conversion tool (Table I) (21). As nifedipine is metabolized mainly via CYP3A and undergoes pre-systemic metabolism, the extent of pre-systemic metabolism was fitted to match the observed AUC of nifedipine following oral administration of 5 mg to fasted healthy subjects (41). Complete absorption was predicted for each dose level (5 mg, 10 mg, and 20 mg), which was in line with reported data (42,43).

Following successful model verification, the pharmacokinetics of the 10 mg nifedipine IR capsule were simulated under fed conditions. Predicted/observed AUC, *C*_max_, and *T*_max_ ratios were 1.51, 1.06, and 1.00, and thus within 2-fold of observed. As in the fasted state, absorption of nifedipine under fed conditions was predicted to be complete.

Additional examples of bottom-up with successful prediction of food effect risk without the need for optimization with clinical food effect data included etoricoxib (negative FE), fluoxetine (no FE), and zidovudine (negative FE). Detailed descriptions of the modeling approach and outcome for these compounds are provided in an accompanying manuscript published in this issue.

### PBPK Predictions to Waive Food Effect Studies—Middle-Out Application

#### Case Example: Aprepitant (High Confidence)

Aprepitant is a poorly soluble compound with moderate to high permeability and is non-ionized at intestinal pH values. Micronized aprepitant showed significant positive food effect (2.8-fold increase in AUC, 2.2-fold increase in *C*_max_) in healthy human volunteers given a high-fat breakfast.

The bottom-up absorption model for the micronized formulation was built according to working group guidelines. Intrinsic and biorelevant solubility data of thermodynamically stable form and micronized drug substance were taken from literature as the drug substance was not commercially available and data was generated using the same approach as described above (13,14). Partitioning of neutral and ionic species into bile micelles (i.e., logK_m:w_) and DLM scalars were estimated using SIVA (14,44,45). Clearance and disposition parameters were estimated from IV PK data (46).

The model was used to simulate oral PK of 100 mg micronized aprepitant in fasted subjects. When the solubility measured in US Pharmacopeia Simulated Intestinal Fluids (SIFsp pH 6.8, 0.7 μg/ml) was used as intrinsic solubility for simulations, PK parameters for the fasted condition were underpredicted (AUC predicted (3354 ng.hr./ml) vs observed (8571 ng.h/ml), *C*_max_ predicted (125 ng/ml) vs observed (496 ng/ml). Therefore, an average of solubility values reported for thermodynamically stable form in water at pH 8.0 (7 μg/ml) and in SIF_sp_ was used as intrinsic solubility which allowed for the prediction of AUC and *C*_max_ values within the pre-specified tolerance (i.e., AUC predicted (11,142 ng.h/ml) vs observed (8571 ng.h/ml), *C*_max_ predicted (455 ng/ml) vs observed (496 ng/ml)). The model was subsequently used to predict the effect of food on PK of a 100 mg micronized aprepitant. The predicted increase in AUC and *C*_max_ was within 0.80–1.25-fold of the observed values (AUC predicted (26,673 ng.h/ml) vs observed (24,057 ng.h/ml), *C*_max_ predicted (1237 ng/ml) vs observed (1098 ng/ml)) (44). Overall, the PBPK model was able to predict the observed positive food effect due to enhanced solubilization and dissolution.

Additional examples with the successful prediction of food effect risk using a middle-out approach include furosemide (negative FE), nefazodone (negative FE), nelfinavir (positive FE), and phenytoin (positive FE). Detailed descriptions of the modeling approach and outcome for these compounds are provided in an accompanying manuscript published in this issue.

### Food Effect Predictions with Low Confidence

#### Case Example: Pazopanib

Pazopanib is a BCS class II/IV compound (basic pKa 2.1 and 6.4) that exhibits low solubility across the physiological pH range. Clinically, pazopanib is dosed as the hydrochloride salt. At 800 mg dose, fasted bioavailability was low and variable at ~ 21% (range 14–39%) and the compound exhibited a significant positive food effect (2.3-fold increase in AUC_0-72h_ and 2.1-fold increase in *C*_max_ with a high-fat meal) (47). The compound is recommended to be taken without food (at least 1 h before or 2 h after a meal) (48).

Bottom-up absorption models were developed following the standardized workflow. Human permeability was projected based on Caco-2 data (Table I). Bile salt solubilization was estimated based on FaSSIF and FeSSIF data. The models (regardless of the software used) significantly underpredicted the fasted state plasma concentration profiles and did not predict the positive food effect. Both bottom-up models underpredicted the fasted state AUC at 800 mg pazopanib-HCl by more than 2-fold. When the models were applied to predicting the effect of food on pazopanib’s PK, they predicted a slight FE (AUC ratio fed/fasted = 0.86 to 1.09), whereas the observed FE was approximately 2.3-fold. Further model optimization in accordance with the decision tree (Fig. 2) by adjusting the precipitation rate constant (essentially reducing *in vivo* precipitation) resulted in closer prediction of the fasted state profile (within 2-fold of AUC). However, the adjusted model failed to predict the significant positive food effect. Models predicted either no food effect or negative food effect, depending on the software used and the stomach pH settings. For both models, the default fed physiology was applied. Thus, the standardized optimization workflow failed to directionally replicate the observed food effect.

The inability to capture the in vivo pharmacokinetics of pazopanib may be due to challenges with modeling dissolution of salts that may be dissolving faster and/or to a greater extent than what the solubility measurements using standard methodologies suggest. Additionally, exploratory experiments suggest that the buffer species has a significant impact on the solubility and precipitation behavior of pazopanib (49). Dissolution measurements in FaSSIF and FeSSIF exhibited some supersaturation followed by precipitation. However, the translation of such *in vitro* observations to a PBPK setting is not straightforward. Additional discussion of pazopanib modeling as related to future opportunities in *in vitro* assays and model refinement are outlined below and a more detailed description of the modeling approach and outcome for pazopanib and trospium chloride is provided in an accompanying manuscript published in this issue.

## DISCUSSION

In this study, a unique prospective approach was proposed to build and verify mechanistic PBPK models for 30 compounds while controlling for common variables, such as model input parameters, method of data generation, and subjective optimization and/or verification. To focus the model development and verification around absorption parameters, compounds with known IV clearance were used. However, we believe in practice the models can be applied even in the absence of IV data if the food effect mechanism is primarily related to absorption events. The focus of this work was to identify mechanisms where high, moderate, and low confidence in the prediction of food effect can be expected. The levels of confidence were defined based on modeling approach and an assessment of how well the PK parameters and profiles were captured. In total, the food effect of 15 compounds was predicted with high confidence, 8 with moderate confidence, and 7 with low confidence. Areas of high to moderate confidence were mainly associated with food effect related to changes in GI luminal fluid and physiology, while lower confidence was commonly associated with complex mechanisms and/or interplay between multiple mechanisms for which standardized *in vitro* assays and model input are not available to characterize the food effect, as well as to develop and verify models and/or gaps exist in capturing these mechanisms in the modeling software utilized.

Compound types where food effect was not appropriately captured using the standardized approach proposed in this work were salts and some weak bases, for which optimizing the precipitation time of the drug could not address the low confidence in capturing the absorption profile and extent of food effect. One key point for the misprediction of food effect is the current gaps in software and/or biorelevant media to adequately reflect the impact of food and food components for poorly soluble drugs; while biorelevant media are widely accepted for providing a good estimate of the luminal solubility of poorly soluble drugs under fasted and fed state, there seem to be cases where the *in vitro* solubility of these media is not in line with the solubility in human intestinal fluids (50), and cases where the buffer species has a pronounced impact on the *in vitro* solubility and precipitation behavior of certain compounds (49). Specifically, the outcome of our modeling exercise suggests that the *in vivo* solubility of pazopanib in the GI tract may be higher compared with the measured *in vitro* FaSSIF solubility. Therefore, there is a need to further investigate more biorelevant media to improve PBPK-based absorption predictions at various prandial states.

In a typical clinical development paradigm, early assessment of food effect could be generated as early as the first-in-human single ascending dose studies. In oncologic drug development, dosing with food (typically a light meal) may be pursued from the beginning, particularly if an increase in exposure is needed or GI-related toxicity can be mitigated. Under current global regulatory paradigms, most companies are expected to re-characterize the food effect on the to-be-marketed formulation. Tistaert *et al.* previously proposed that following validation of the model against fasted and fed data that have been generated in early development, one could apply the model prospectively to account for food effect of new doses, formulations, or API forms (9). In this manuscript, we have built on these recommendations by highlighting opportunities where we find the translation of these models across formulations may be appropriate and where *in vitro* methodology and PBPK models may require further advancements in their approach to be adequately predictive. When the mechanisms of food effect can be categorized as high confidence, bottom-up, the PBPK models may be used for decision-making around food effect prior to FIH and also, it may not be required to perform a clinical anchor study (i.e., a specific food effect study in healthy volunteers under well-controlled conditions and powered to show bioequivalence). In such cases, the high confidence PBPK FE projections could still be confirmed with clinical data (e.g., an arm of fed subjects in a Ph1 study or a PopPK analysis of patient data) (51). It is also worth noting that the relevant food effect in clinical use in the patient population may differ from that measured in an anchor study in healthy volunteers with a standard meal. Such drugs may include compounds where solubility is generally not rate-limiting and food effect (typically negative for *C*_max_) is primarily dictated by gastric emptying.

Mechanisms categorized as high confidence, middle-out may need to be verified using an anchor study before being used to predict food effect with high accuracy. However, there is still the possibility to avoid additional clinical studies around food effect after certain formulation or dose changes. Similarly, when models and mechanisms categorized as having moderate confidence (within 2-fold) are verified with an anchor study, there may be cases where minor formulation changes can be made without the need for additional FE studies. At this point, the nature of the formulation change should be considered. Formulation changes are commonplace during clinical development. While, on some occasions, major formulation changes may be pursued to address specific clinical needs (e.g., an “enabled” formulation to address the poor bioavailability of an early crystalline formulation), most formulation changes undertaken are more subtle. The formulation technology is typically decided early on and optimization focuses on the composition and manufacturing process. Based on the work presented in this manuscript, we propose that if the formulations follow the same dissolution/absorption principles (e.g., both formulations are based on crystalline API), translation of the models from early to late stage is possible especially when the mechanism of food effect (or lack thereof) is well understood (i.e., the defined confidence categories should hold). For many of the examples highlighted in this work, the formulation dissolution rate is not the determining factor for food effect. For rapid-dissolving formulations, food effect may be dictated by gastric emptying time (if sufficient solubility or administered as non-precipitating oral solution) or by the saturation solubility of the API in the fasted and fed condition. This is mostly independent of the formulation and would not be affected by many formulation changes. However, the authors acknowledge that the proposal to avoid clinical studies for minor formulation changes has been supported by only limited published examples (9,52) and further work is recommended to verify this with a broader range of clinical data. While the primary application of such models is to describe the food effect in the context of biopharmaceutics characterization, on a case-by-case basis, these models can be considered for prospective application dependent on therapeutic margins and exact mechanism of food effect verified with the anchor study.

Like formulation changes, we believe that PBPK models could be applied to the projection of food effect at different doses than previously established. A properly qualified PPBK model across different doses would accurately account for absorption limitations (if applicable) and thus would be well-positioned to assess the impact of food at different doses than previously studied. An interesting question would be the application of food effect models to different meals (e.g., a moderate-fat, moderate-calorie meal). There are currently a relatively limited number of published examples systematically looking at parameterization of the models as a function of the meal content. The most detailed example is probably the study by Sutton *et al.* (53) where the authors proposed adjustments to the gastric emptying and bile salt concentration settings in the PBPK model for different meals. Adopting such a model may be considered to interpolate exposures between the typically tested prandial states (i.e., fasted and high-fat/high-calorie meal). While this could help with simulating more real-life dosing conditions, it was out of scope of the work presented here.

Food effect prediction with a PBPK model seamlessly integrates *in vitro* data with knowledge of human physiology. Prior work has attempted food effect prediction from only *in vitro* data (54–56) or directly from animal models (56–58). While these methods have shown some success in predicting the direction of food effect, their ability to accurately capture the extent of food effect, and the impact of food on PK parameters and, especially, profiles have not been consistently demonstrated. While it makes sense to use all available data, including pre-clinical data (e.g., dog FE data), to inform predictions of human food effect, the focus of this study was to explore the predictive ability of PBPK models following a pure in vitro to in vivo approach. We present the application of standard PBPK platforms to accurately capture not only the direction of food effect but also the extent of the effect on the human PK profile. Given the complex nature of food effect, it is anticipated that an integrated approach such as PBPK, which captures the complexity of human physiology and disparate food effect mechanism, can serve as a key platform for the support of food effect predictions.

The study of food effect on the absorption of orally administered drugs is widespread and a key driver of study design, data analysis, and labeling language. Within the pharmaceutical industry, significant resources are invested to predict and characterize a clinically meaningful food effect, including the use of PBPK models to gain mechanistic insight into potential food effect. Here, the predictive performance of PBPK food effect models was assessed using *de novo* mechanistic absorption models for 30 compounds generated in accordance with controlled, pre-defined in vitro and modeling methodology, as well as an aligned decision tree for model design, verification, and optimization. Mechanistic PBPK models enabled food effect modeling of most of the compounds with high (50%) or moderate (27%) confidence, with a small subset of compounds showing low (23%) confidence in the prediction of food effect. A correlation was observed between the confidence in the model and the mechanism of food effect, whereby models generally showed high confidence in prediction where food effect was related to changes in GI fluid volume, motility, or luminal fluid composition, while food effects related to drug interactions with intestinal microenvironment and/or food-drug/micelle-drug interactions were more difficult to predict with high confidence. This analysis did not include prodrugs and compounds whose absorption is known to be limited by intestinal active transport; however, such compounds are not expected to make up a significant subset of clinical compounds displaying food effect. While the correlation between model confidence and mechanism of food effect was established for 30 compounds in this study, it is only the first step in understanding this correlation. Future work should focus on further strengthening the validity of these conclusions by expanding this analysis to additional compounds.

## CONCLUSION

Considering these findings, it is recommended that appropriately verified, mechanistic PBPK models be leveraged as a key approach to studying potential food effect, especially related to mechanisms associated with high to moderate confidence, thereby replacing the need to conduct clinical food effect studies.

## Electronic supplementary material

ESM 1 (DOCX 53 kb)

ESM 2 (ENL 4 bytes)
