# Supplementary material for: Use of Physiologically Based Pharmacokinetic (PBPK) Modeling for Predicting Drug-Food Interactions: an Industry Perspective
Source: AAPS J. 2020 Sep 27;22(6):123. doi: 10.1208/s12248-020-00508-2 (PMC7520419; doi:10.1208/s12248-020-00508-2)
Supplement: Supplementary file 1 — (DOCX 53 kb) [file 12248_2020_508_MOESM1_ESM.docx]

# Supplemental Material

**Supplementary Table 1 – List of all compiled compounds with food effect.** The list was narrowed down to 30 compounds for PBPK model analysis by the working group based on various exclusion criteria and exceptions described below. Green columns indicate the compounds that were selected for analysis.

| **Compound** | **Food Effect** | **Included in WG Analysis (Yes/No)** | **Reason for Exclusion** |
| --- | --- | --- | --- |
| 5-Aminosalicylic acid | Negative | No | Food effect conducted with IR suspension, which was not available to working group for data generation |
| Abacavir sulfate | None | No | Food effect conducted with a formulation that was not available to working group for data generation |
| Acitretin | Positive | No | Lacking key in vitro or formulation information |
| Acyclovir | None | No | Prodrug (exclusion criteria) |
| Albendazole | Positive | No | Lacking IV PK data (exclusion criteria); absorption potentially limited by transport (exclusion criteria) |
| Albuterol (Salbutamol) | None | No | Food effect study conducted with MR formulation, not available to working group for data generation |
| Alectinib | Positive | Yes | Not applicable |
| Alfuzosine | Positive | No | Insufficient detail on food effect study in the literature |
| Amiodarone | Positive | Yes | Not applicable |
| Aprepitant | Nanonized: None;  Micronized: Positive | Yes | Not applicable |
| Aripiprazole | None | No | Absorption potentially limited by transport (exclusion criteria) |
| Atazanavir sulfate | Positive (low fat); Negative (high fat) | No | Absorption potentially limited by transport (exclusion criteria) |
| Atorvastatin | None | No | Absorption potentially limited by transport (exclusion criteria) |
| Atovaquone | Positive | No | Lacking information on formulation and particle size from the food effect study |
| Axitinib | None | No | Absorption potentially limited by transport (exclusion criteria) |
| Bicalutamide | None | No | Lacking IV PK data (exclusion criteria) |
| Bidisomide | Negative | No | No drug product available to working group for data generation |
| Bromazepam | Negative | No | Lacking IV PK data (exclusion criteria) |
| Bromocriptine | None | No | Lacking key in vitro or formulation information |
| Cabozantinib | Positive | No | Lacking key in vitro or formulation information |
| Capecitabine | Negative | No | Food effect data available only in patients |
| Captopril | Negative | No | Lacking IV PK data (exclusion criteria) |
| Cefdinir | None | No | Lacking IV PK data (exclusion criteria) |
| Cefditoren pivoxil | Positive | No | Prodrug (exclusion criteria) |
| Ceftibuten dihydrate | Negative | No | Lacking key in vitro or formulation information |
| Cefuroxime axetil | Positive | No | Prodrug (exclusion criteria) |
| Celecoxib | Slightly positive | No | Lacking IV PK data (exclusion criteria) |
| Chlorothiazide | Positive | No | Lacking key in vitro or formulation information |
| Cimetidine | None | Yes | Not applicable |
| Cinnarizine | Positive | No | Lacking IV PK data (exclusion criteria) |
| Ciprofloxacin | None | No | Absorption potentially limited by transport (exclusion criteria) |
| Clarithromycin | Positive | Yes | Not applicable |
| Clodronate | Negative | No | Food effect conducted with complex formulation, which was not available to working group for data generation |
| Clopidogrel bisulfate | Positive (prodrug); None (active metabolite) | No | Prodrug (exclusion criteria) |
| Crizotinib | None | No | Absorption potentially limited by transport (exclusion criteria) |
| Dabrafenib | Negative | Yes | Not applicable |
| Danazol | Positive | Yes | Not applicable |
| Danirixin | Negative | Yes | Not applicable |
| Didanosine | Negative | No | Lacking key in vitro or formulation information |
| Dolasetron mesylate | None | No | Lacking key in vitro or formulation information |
| d-Sotalol | None | Yes | Not applicable |
| Efavirenz | Positive | No | Lacking IV PK data (exclusion criteria) |
| Entecavir | Negative | No | Lacking IV PK data (exclusion criteria) |
| Eptastigmine | Negative | No | Lacking IV PK data (exclusion criteria) |
| Erlotinib | Positive | No | Lacking key in vitro or formulation information |
| Ethinyl estradiol | None | No | Lacking IV PK data (exclusion criteria) |
| Etoricoxib | Negative | Yes | Not applicable |
| Fenofibrate | Positive | No | Prodrug (exclusion criteria) |
| Fenoldopam | Negative | No | Lacking key in vitro or formulation information |
| Fexofenadine | Negative | No | Lacking IV PK data (exclusion criteria); Absorption potentially limited by transport (exclusion criteria) |
| Fluoxetine hydrochloride | None | Yes | Not applicable |
| Frusemide (Furosemide) | Negative | Yes | Not applicable |
| Ganciclovir | None | No | Lacking key in vitro or formulation information |
| Gefitinib | Positive | No | Lacking key in vitro or formulation information |
| Griseofulvin | Positive | No | Lacking IV PK data (exclusion criteria) |
| Halofantrine hydrochloride | Positive | No | Lacking IV PK data (exclusion criteria) |
| Hydralazine | Negative | No | Food effect mechanism related to extensive first pass (exclusion criteria) |
| Hydrochlorothiazide | None | No | Lacking IV PK data (exclusion criteria) |
| Ibrutinib | Positive | No | Lacking IV PK data (exclusion criteria) |
| Imatinib | None | Yes | Not applicable |
| Imiquimod | None | No | Lacking IV PK data (exclusion criteria) |
| Indinavir | Negative for free base, no food effect for salt | No | Absorption potentially limited by transport (exclusion criteria) |
| Irbesartan | None | No | Lacking key in vitro or formulation data |
| Isoniazid | Negative | Yes | Not applicable |
| Isosorbide Mononitrite | None | No | Lacking key in vitro or formulation information |
| Isotretinoin | Positive | No | Lacking key in vitro or formulation information |
| Itraconazole | Positive | Yes | Not applicable |
| Ivacaftor | Positive | Yes | Not applicable |
| Lamivudine | None | No | Lacking key in vitro or formulation information |
| Lansoprazole | None | No | Lacking key in vitro or formulation information |
| Lapatinib | Positive | No | Absorption potentially limited by transport (exclusion criteria) |
| Lomefloxacin | None | No | Lacking IV PK data (exclusion criteria) |
| Mefloquine hydrochloride | Positive | No | Lacking IV PK data (exclusion criteria) |
| Meloxicam | None | No | Lacking key in vitro or formulation information |
| Metformin | None | No | Absorption potentially limited by transport (exclusion criteria) |
| Metoprolol | Positive | Yes | Not applicable |
| Misoprostol | None | No | Lacking IV PK data (exclusion criteria) |
| Nefazodone hydrochloride | Negative | Yes | Not applicable |
| Nelfinavir mesylate | Positive | Yes | Not applicable |
| Nifedipine | None | Yes | Not applicable |
| Ofloxacin | None (Absorption delayed but extent not substantially affected) | No | Lacking key in vitro or formulation information |
| Oseltamavir | None | Yes | Not applicable |
| Panobinostat | None | Yes | Not applicable |
| Pazopanib | Positive | Yes | Not applicable |
| Phenytoin | Positive | Yes | Not applicable |
| Posaconazole | Positive | No | Used as a test case; not included in the analysis |
| Progesterone | Positive | No | Lacking key in vitro or formulation information |
| Ribavirin | Positive | No | Food effect data available only in patients |
| Regorafenib | Positive | No | Lacking IV PK data (exclusion criteria) |
| Ritonavir | None | No | Absorption potentially limited by transport (exclusion criteria) |
| Rofecoxib | None | No | Lacking IV PK data (exclusion criteria) |
| Rosuvastatin | Negative | No | Absorption limited by transport (exclusion criteria) |
| Ruxolitinib | None | No | Lacking IV PK data (exclusion criteria) |
| S(+)-Ibuprofen | None | No | Lacking key in vitro or formulation information |
| Saquinavir | Positive | No | Transporter substrate (exclusion criteria) |
| Sarpogrelate | Negative | No | Lacking IV PK data (exclusion criteria) |
| Sertraline | None | No | Lacking IV PK data (exclusion criteria) |
| Sitagliptin | None | No | Lacking key in vitro or formulation information |
| Sonidegib | Positive | No | Lacking key in vitro or formulation information |
| Tafinlar | Negative | No | Absorption limited by transport (exclusion criteria) |
| Tamsulosin hydrochloride | Negative | No | Lacking data for model verification |
| Telapravir | Positive | Yes | Not applicable |
| Temafloxacin HCl | None | No | Lacking key in vitro or formulation information |
| Tezacaftor | None | Yes | Not applicable |
| Ticlopidine hydrochloride | Positive | No | Lacking key in vitro or formulation information |
| Tolectin (tolmetin) | Negative | No | Lacking key in vitro or formulation information |
| Tolterodine tartrate | None | No | Lacking key in vitro or formulation information |
| Topiramate | None | No | Lacking key in vitro or formulation information |
| Triclabendazole | Positive | No | Lacking IV PK data (exclusion criteria) |
| Troglitazone | Positive | No | Drug has been discontinued |
| Trospium Chloride | Negative | Yes | Not applicable |
| Valdecoxib | None | No | Food effect conducted with formulation, which was not available to working group for data generation |
| Vemurafenib | Positive | No | Lacking IV PK data (exclusion criteria) |
| Venetoclax | Positive | Yes | Not applicable |
| Verapamil hydrochloride | None | No | Food effect conducted with formulation, which was not available to working group for data generation |
| Zalcitabine | None | No | Drug has been discontinued |
| Zidovudine | Negative | Yes | Not applicable |
| Ziprasidone hydrochloride | Positive | Yes | Not applicable |
| Zolmitriptan | None | No | Lacking key in vitro or formulation information |
